# Identification of transposon insertion mutants of *Francisella tularensis tularensis *strain Schu S4 deficient in intracellular replication in the hepatic cell line HepG2

**DOI:** 10.1186/1471-2180-6-69

**Published:** 2006-07-31

**Authors:** Aiping Qin, Barbara J Mann

**Affiliations:** 1 Department of Internal Medicine, Division of Infectious Diseases and International Health, University of Virginia, Charlottesville, VA 22908, USA; 2 Department of Microbiology, University of Virginia, Charlottesville, VA 22908, USA

## Abstract

**Background:**

*Francisella tularensis *is a zoonotic intracellular bacterial pathogen that causes tularemia. The subspecies *tularensis *is highly virulent and is classified as a category A agent of biological warfare because of its low infectious dose by an aerosol route, and its ability to cause severe disease. In macrophages *F. tularensis *exhibits a rather novel intracellular lifestyle; after invasion it remains in a phagosome for three to six hours before escaping to, and replicating in the cytoplasm. The molecular mechanisms that allow *F. tularensis *to invade and replicate within a host cell have not been well defined.

**Methods:**

We constructed a stable transposon mutagenesis library of virulent strain Schu S4 using a derivative of the EZ::TN transposon system^®^. Approximately 2000 mutants were screened for the inability to invade, and replicate in the hepatic carcinoma cell line HepG2. These mutants were also tested for replication within the J774.1 macrophage-like cell line.

**Results:**

Eighteen mutants defective in intracellular replication in HepG2 cells were identified. Eight of these mutants were auxotrophs; seven had mutations in nucleotide biosynthesis pathways. The remaining mutants had insertions in genes that were predicted to encode putative transporters, enzymes involved in protein modification and turnover, and hypothetical proteins. A time course of the intracellular growth of a *pyrB *mutant revealed that this mutant was only able to grow at low levels within HepG2 cells but grew like wild-type bacteria in J774.1 cells. This *pyrB *mutant was also attenuated in mice.

**Conclusion:**

This is the first reported large-scale mutagenesis of a type A strain of *F. tularensis *and the first identification of mutants specifically defective in intracellular growth in a hepatic cell line. We have identified several genes and pathways that are key for the survival and growth of *F. tularensis *in a hepatic cell line, and a number of novel intracellular growth-defective mutants that have not been previously characterized in other pathogens. Further characterization of these mutants will help provide a better understanding of the pathogenicity of *F. tularensis*, and may have practical applications as targets for drugs or attenuated vaccines.

## Background

*Francisella tularensis *is a gram-negative bacterium that causes a potentially life-threatening disease called tularemia [1]. There are several subspecies of *Francisella *but *F. tularensis tularensis *and *F. tularensis holarctica*, also known as Type A and Type B, respectively, are the most prevalent. Type A isolates have been classified as a category A select agent of biological warfare. They are typically restricted to North America, and can cause a severe infection that is potentially fatal, particularly if untreated. Type B isolates are found primarily in the Northern Hemisphere, and cause a milder disease that is rarely fatal. These bacteria are zoonotic organisms that can infect hundreds of different species. *F. tularensis *is usually transmitted to humans, an incidental host, by the bite of an arthropod vector but it is also possible to become infected through an aerosol route.

*F. tularensis *is a facultative intracellular bacterium that can invade a variety of cell types including macrophages, endothelial cells, and hepatocytes [2-4]. In macrophages *F. tularensis *exhibits a rather unusual intracellular lifestyle. Most intracellular bacteria reside and replicate within a remodeled phagosome that is able to avoid lysosomal fusion through a variety of mechanisms. Other bacteria rapidly escape the phagosome after invasion and replicate directly in the cytoplasm. *Francisella *seems to have combined these two pathways; after uptake it remains in a phagosome for three to six hours before escaping to, and replicating in the cytosol [5-8]. A few factors have been identified that regulate or facilitate phagosome escape and intracellular survival of *F. tularensis *[9-12], but relatively little is known about the molecular mechanisms or necessary requirements of this intracellular lifestyle. To gain a better understanding of the requirements for invasion, phagosome escape, and intracellular replication and survival, we created a transposon mutagenesis library of the type A strain Schu S4 using EZ::TN <*rpsL*^*p*^*Rparr-2*>, a derivative of the EZ::TN transposon system^® ^[13]. Schu S4 is a human isolate, and its genome has been completely sequenced [14]. Approximately 2000 mutants were screened for the inability to replicate in the hepatic cell line, HepG2. This cell line was chosen because mouse studies of tularemia have indicated that the liver is the primary site of tularemia pathology, and intracellular bacteria are visible in hepatocytes [15]. The range of mutants isolated in this screen has identified some key aspects of the requirements for intracellular survival in a hepatic cell line. Further characterization of these mutants will help to define the pathways, and processes that lead to successful infection.

## Results

### Construction of a transposon-insertion library of Schu S4

**Figure 1:**
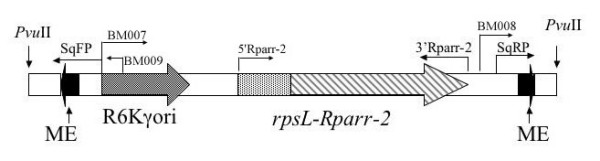
**Map of transposon EZ::TN<*rpsL*^*p *^*Rparr-2*> that was used to create transposon-insertion library in Schu S4**. *Rparr-2 *is a gene that encodes resistance to rifampin by inactivation of the drug. Arrows indicate the location of various primers that were used to for PCR amplification of the transposon or sequencing the flanking DNA. ME-mosaic ends (specifically recognized by the EZ::TN transposase), R6Kγori is an origin of replication that is used to rescue the transposon after it is inserted into the genome.

We used the transposon EZ::TN<*rpsL*^*p*^*Rparr-2*> to create a transposon-insertion library of the *F. tularensis *type A strain Schu S4 (Fig. 1). This transposon was previously used to identify mutants in *Rickettsia prowazekii *and carries the gene *Rparr-2*, which encodes an enzyme that inactivates rifampin by ADP-ribosylation [13]. The transposon was isolated from pMW1409 by restriction enzyme digestion and gel electrophoresis. The purified transposon was incubated with transposase, and then the "transposome" complex was introduced into Schu S4 by electroporation. Rifampin-resistant colonies were selected on MHA plates containing rifampin. Approximately 10,000 rifampin-resistance colonies were isolated, individually grown, and frozen in 96 well microtiter plates. The presence of the transposon in the rifampin-resistant colonies was verified by PCR amplification of the transposon from DNA isolated from the mutants using transposon-specific primers BM007 and 3'arr-2 (data not shown). The transposition frequency was approximately 2.0 × 10^-5 ^per input colony forming unit (CFU).

**Figure 2:**
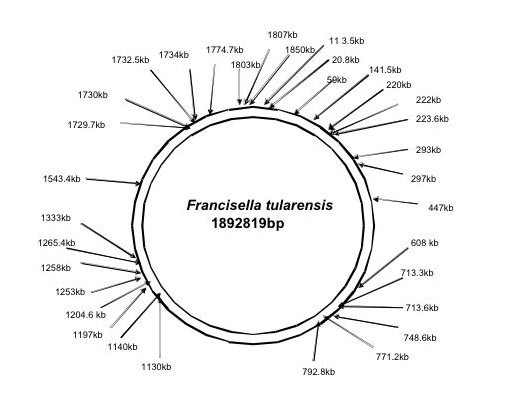
**Chromosomal location of transposon EZ::TN<*rpsL*^*p *^*Rparr-2*> in selected strains from the transposon-insertion library of Schu S4**. A circular map of the Schu S4 chromosome showing the location of transposon insertion sites

To examine the randomness of the transposition event the transposon with its flanking DNA was rescued from 33 colonies by digesting the DNA with *Bcl1*, self-ligating the fragments, and introducing them into *pir*^+^*E. coli*. The DNA flanking the transposon was sequenced, and found to be 100% identical to Schu S4 sequences. The location of each transposon insertion site on a chromosomal map of Schu S4 is shown in Fig. 2. Two insertion sites appeared to be in intergenic regions while the rest were within putative open reading frames. The insertion sites were generally spread around the chromosome with no major hotspots, however there were three regions of significant size to which no insertion sites mapped.

To test the stability of the transposition event ten mutants were replated five times on MHA with or without rifampin. In all cases rifampin resistance was stable. In two strains the DNA flanking the transposon was sequenced, and found to be identical to that of the original rescued plasmid. These results indicated that the transposon was stable once integrated into the Schu S4 genome.

### Isolation of mutants defective in intracellular survival in HepG2 cells

**Figure 3:**
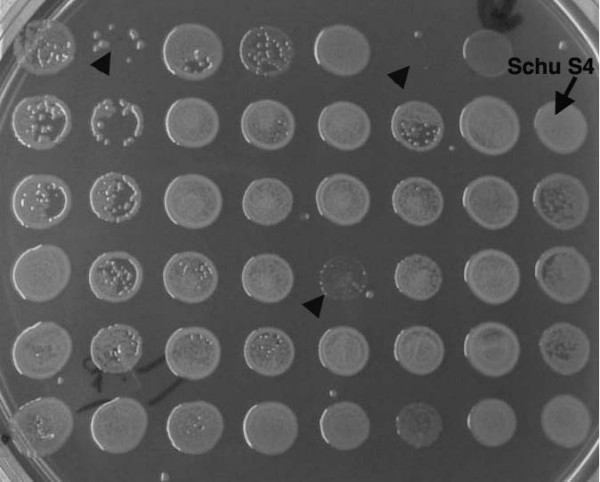
**Identification of mutants defective in intracellular growth in HepG2 cells**. Individual transposon-insertion mutants were incubated with HepG2 cells for two hours, treated with gentamicin, incubated for an additional 24 hrs, then the HepG2 cells were lysed. The lysates were transferred to MHA plates with a 48-pin replicator and incubated for 48 hours. Representative mutants that showed no or reduced growth after 48 hrs are indicated by arrowheads.

**Figure 4:**
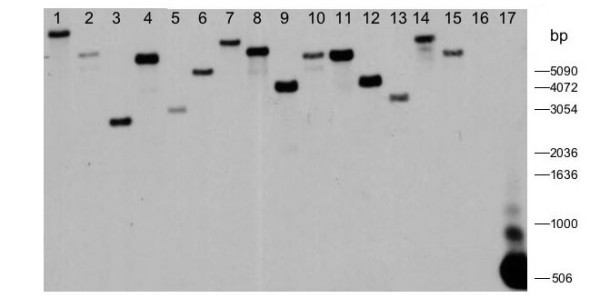
**Southern blot of transposon insertion mutants of Schu S4 that were defective in intracellular growth in HepG2 cells**. Chromosomal DNA from mutants digested with BclI was subjected Southern hybridization with a PCR product amplified from the EZ::TN<*rpsL*^*p*^*Rparr-2*> DNA template. Lanes 1–15 contain DNA from rifampin-resistant mutants of Schu S4. Lane 16 contains wild-type Schu S4 DNA and Lane 17 contains the probe DNA. Each mutant contains a single copy of the transposon.

To identify potential virulence factors, and also define requirements for intracellular survival of Schu S4, the transposon-insertion library was screened for mutants defective in intracellular survival. We screened 1,974 independent mutants by incubating each mutant with HepG2 cells in a microtiter well. After two hours the cells were washed, and gentamicin was added. After a 24 hr incubation the cells were lysed, and each lysate was transferred to a MHA plate and further incubated for 48 hours (Fig. 3). Strains that failed to grow on the MHA plate were retested two more times. Twenty-seven mutants were finally identified that failed to replicate within HepG2 cells. The location of the transposon insertion site in the Schu S4 genome for each mutant was determined (Table 2). Eighteen different genes were identified from these 27 mutants. In all cases the transposon inserted in orientation such that the direction of transcription of the *Rparr-2 *gene was the same as the disrupted open reading frame. This suggests that the *Rickettsia rpsL *promoter was not active in Schu S4, and that the expression of the *Rparr-2 *gene was driven by the nearest upstream promoter. A Southern blot of DNA isolated from 15 of these strains, and hybridized with a probe specific for *Rparr-2*, demonstrated that each mutant had a single integrated copy of EN::TN<*rpsL*^*p*^*Rparr-2*> (Fig. 4). Fifteen of the 18 genes disrupted by a transposon had annotations for a predicted gene function (Table 2). Only three were designated as hypothetical. In several of these mutants the transposon had inserted into a gene that appeared to be part of an operon, therefore the defect in intracellular survival in these mutants may be due to a polar effect on downstream genes. Eight of the 18 mutants were auxotrophs. Seven of these 8 auxotrophic mutants had transposon insertions in genes involved in purine or pyrimidine nucleotide biosynthesis.

### Characterization of a mutant in the pyrimidine nucleotide biosynthesis pathway

**Figure 5:**
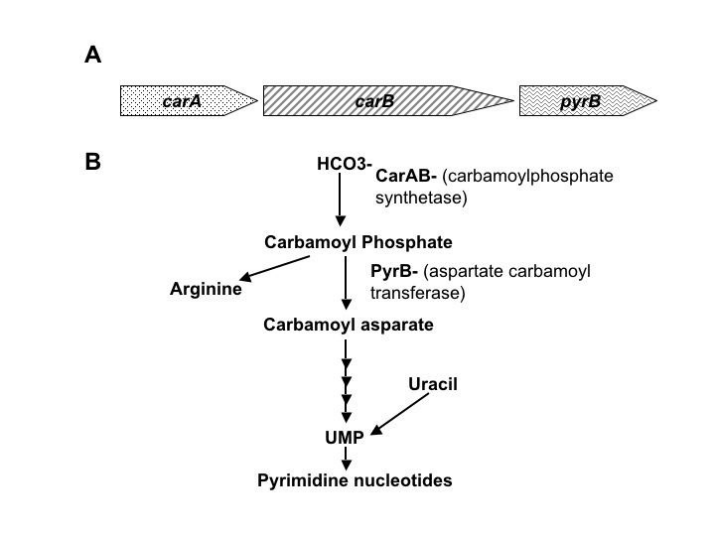
**Predicted operon (5A) and simplified pyrimidine nucleotide synthesis pathway (5B) in *F. tularensis***. The direction of transcription of the putative operon is from left to right.

**Figure 6:**
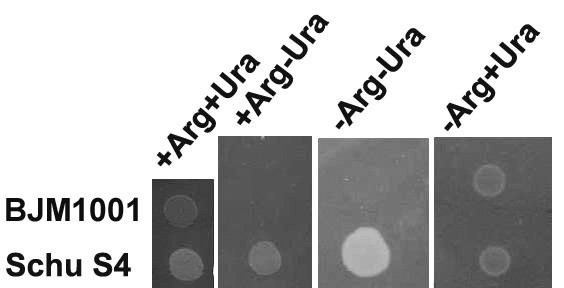
**Uracil auxotrophy of *pyrB *mutant BJM1001**. Schu S4 mutant BJM1001 with transposon insertion in *pyrB *was spotted on CDM minimal media, containing supplements as indicated, and grown for 72 hours.

**Figure 7:**
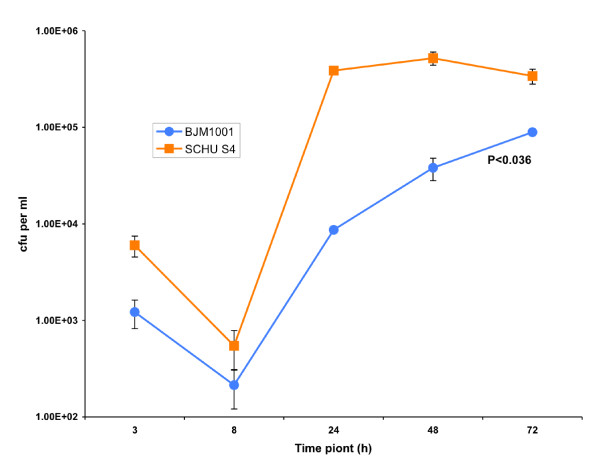
**Invasion and intracellular survival of BJM1001 and Schu S4 in HepG2 cells**. HepG2 cells were infected with BJM1001 at an MOI to 50:1 in microtiter wells. After 2 hrs the media was replaced with media containing gentamicin. Sets of three wells were lysed at 3, 8, 24, 48 and 72 hrs, diluted, and spread on MHA plates to determine the number of intracellular colony forming units (CFUs). Each time point represents the average and standard deviation (S.D.) of three wells.

The initial enzymes in the pyrimidine nucleotide biosynthesis pathway appear to be encoded by a three-gene operon consisting of *carA, carB*, and *pyrB *(Fig. 5). The products of these genes are carbamoyl-phosphate synthase (small and large subunit), and aspartate carbamoyltransferase. The phenotype of BJM1001 (*pyrB*) was characterized in greater detail. *B. abortus pyrB *mutants are defective in intracellular growth in macrophages though these bacteria reside within phagosomes [16]. As expected, BJM1001 required added uracil, and not arginine for growth on minimal CDM (Fig. 6). The ability of BJM1001 to invade and replicate in HepG2 was compared to wild-type Schu S4 (Fig. 7). At three hours post infection there were approximately one third fewer intracellular CFUs of BJM1001 than Schu S4. At 24 hours the number of CFUs of Schu S4 had increased more than 1000 fold while BJM1001 numbers increased approximately 50 fold. At 48 hours the number of Schu S4 bacteria remained roughly constant and BJM1001 had again about a 50-fold increase. At 72 hrs the number of Schu S4 had begun to decline, while BJM1001 continued to slowly increase. In summary, the growth defect of BJM1001 in HepG2 cells manifested early in infection, however the bacteria continued to grow, albeit at low levels, for at least 72 hrs post-infection.

The ability of BJM1001 to invade and replicate in J774.1 cells, a murine macrophage-derived cell line, was also examined. J774.1 cells were infected with bacteria at an MOI to 50:1 in microtiter wells in triplicate. After 2 hrs the media was replaced with media containing gentamicin. The wells were lysed at 3, and 24 hrs, diluted, and spread on MHA plates to determine the number of intracellular CFUs. In contrast to HepG2 cells, intracellular growth of BJM1001 in J774.1 cells was similar to Schu S4. At 3 and 24 hrs the number of CFUs recovered from BJM1001 and Schu S4 infected cells was approximately the same. At 24 hrs the number of intracellular bacteria in both strains had increased by about 3 logs. Although the same number of host cells and MOI were initially used to infect HepG2 and J774.1 cells, the number of CFUs recovered from J774.1 cells at 3 and 24 hrs was nearly 3 logs higher than with HepG2 cells. The experiment with J774.1 cells could not be continued past the 24 hour time point because the J774.1 cells began to die. Several other mutants were tested for intracellular growth in J774.1 cells (Table 3 and data not shown). Among 11 mutants tested, BJM1005 (*dsbB*) and BJM1026 (hypothetical protein) were profoundly defective in intracellular growth in J774.1 cells while two others, BJM1004 and BJM1025, were moderately defective. The mutant strains that grew similarly to Schu S4 in J774.1 cells were BJM1001, BJM1002, BJM1003, BJM1012, BJM1015, BJM1018, and BJM1024. These results suggest that the requirements for intracellular growth, or possibly the intracellular environment of *F. tularensis *in these two cell lines are quite different.

To determine whether the delayed growth phenotype of BJM1001 in HepG2 cells translated to attenuation in vivo, mice were infected with approximately 200 or 2000 CFUs of BJM1001 or Schu S4 by an intranasal route. All mice infected with 2000 CFUs of Schu S4 died or were euthanized on Day 4. All mice infected with 200 CFUs of Schu S4 died or were euthanized on Day 5. Some attenuation of BJM1001 was observed; three out of four mice challenged with 2000 CFUs of BJM1001 died on Day 6, which was a two day delay compared to a similar inoculum of Schu S4. Two out of four mice challenged with 200 CFUs BJM1001 also died on Day 6. The three remaining mice appeared healthy with normal activity until day 10, when they were euthanized. By gross examination the livers of the surviving mice appeared normal, however bacteria were isolated from liver homogenates. These results suggest that the ability to grow within HepG2 cells correlates with in vivo virulence.

## Discussion

*F. tularensis *is remarkable for its ability to infect an array of mammals, arthropods, and even protozoa with an infectious dose of 10 organisms or less [17]. To accomplish these feats, the bacterium must have devised efficient means of infecting its host, and effective ways of avoiding host defense mechanisms. A key component of successful infection should rely at least in part on its ability to invade, survive, and replicate inside host cells. The intracellular lifestyle of *F. tularensis *in macrophages represents a novel paradigm for intracellular survival. Once inside a macrophage *F. tularensis *is able to escape from the phagosome and replicate in the cytoplasm. Unlike other cytosolic intracellular bacteria such as *Shigella sp *and *Listeria monocytogenes*, which escape from their phagosomes within minutes after entering host cells [18,19], *Francisella *resides in phagosomes that acquire endosomal markers such as early endosomal antigen (EEA) 1 and the late endosomal markers (LAMP) 1 and 2, for three to six hrs after infection before escaping into the cytoplasm [5-8,20]. In this study we used a random mutagenesis approach to identify genes in *F. tularensis *that contribute to this novel pathway of intracellular survival and replication.

A stable transposon-mutagenesis library was constructed in Schu S4, a virulent isolate of *F. tularensis tularensis*. Nearly all of the transposon-insertion sites were located within open reading frames. It therefore appears that an upstream endogenous promoter drove the transcription of the transposon-encoded rifampin-resistance gene *Rparr-2 *in Schu S4. Therefore our library is not completely comprehensive because mutations in genes that are poorly expressed in our culture conditions would not have been isolated. We screened our library for mutants defective in intracellular survival in HepG2 cells. A hepatic cell line was chosen because in the mouse tularemia model, regardless of the route of infection, bacteria disseminate to the liver [15]. The genes that were disrupted in these mutants provide insights into the pathways and processes that are required for intracellular replication and survival of *F. tularensis*. We did not identify in our screen any mutants in the 30 kb pathogenicity island in *F. tularensis *that encodes several novel proteins including IglA, IglC, PdpA and PdpB, which are required for intracellular replication and survival of *F. novicida *in macrophages [7,9]. This was not unexpected because this pathogenicity island is duplicated in Schu S4. By chance one of the randomly selected mutants, which was not defective in intracellular growth, had a transposon inserted in *pdpA *(see Fig. 2, insertion at nucleotide 1774.7).

Seven out of the eight auxotrophic mutants identified in our screen had transposon insertions in genes involved in purine or pyrimidine nucleotide biosynthesis. Intact purine or pyrimidine biosynthesis pathways have been found to be important for the virulence of several other intracellular bacteria that reside within phagosomes, including *Brucella abortus *[21], *Brucella suis *[22], *Salmonella typhimurium *[23], and *Mycobacteria tuberculosis *[24]. Purine prototrophy has also been shown to be important for the intracellular survival and virulence of *Shigella flexneri*, a bacterium that replicates in the cytoplasm [25]. In our study the *pyrB *mutant, BJM1001, was defective in intracellular replication in HepG2 cells, though it was able to continue to grow at reduced levels. BJM1001 was also attenuated in mice, exhibiting a delayed time to death when compared to Schu S4. Although this is a single mutant, it suggests that the ability to replicate within HepG2 cells may be useful as a correlative test for virulence in vivo. This mutant showed no growth defects in J774.1 cells. These results suggest that the availability or accessibility of pyrimidines to the bacteria is different in these two cell types. The intracellular fate of *F. tularensis *has really only been examined carefully in macrophages or macrophage cell lines, and not in hepatocytes or hepatic cell lines. The time course of phagosomal escape may be different in hepatic cells or may not even occur. The differences in the ability of some of our mutants to replicate in these two cell types may be a reflection of the exposure of *F. tularensis *to different intracellular compartments or environments, or some species-specific factors since HepG2 cells are a human-derived cell line and J774.1 have a murine origin.

Most intracellular gram-negative bacteria reside within a phagosome, and have developed different mechanisms to avoid fusion with lysosomes. The best-studied exceptions are *Shigella *sp., which survive and replicate directly in the cytoplasm. One explanation for this skew is that the cytoplasm is not a particularly hospitable environment for bacterial growth and survival. Although the intracellular fate of *F. tularensis *in hepatocytes has not been studied in detail, two mutants obtained in our screen, *dsbB *and *ggt*, disulfide bond formation protein B and gamma-glutamyl transferase (GGT), respectively, suggest that *F. tularensis *is in contact with the cytosol in hepatocytes, and may reflect *F. tularensis *adaptation to living in the cytoplasm. The cytosol is a reducing environment due to glutathione reductase, and contains high levels of reduced glutathione, which is necessary to inhibit protein folding in the cytoplasm [25]. The periplasm of gram-negative bacteria typically contain several disulfide bond formation proteins that catalyze disulfide bond exchange and help to maintain the balance of redox potential [26]. DsbA catalyzes disulfide bond formation and DsbB oxidizes DsbA back to its active oxidized form. A functional DsbB appears to be critical for *F. tularensis *intracellular growth because this mutant exhibited one of the most severe growth defective phenotypes in both HepG2 and J774.1 cells.

*DsbA *has been identified as a virulence-associated gene in several other pathogens [27]. DsbA also has a role in the formation of pili. It is required for biogenesis of the type IV pili of the *Vibrio cholerae *toxin-coregulated pilus and the bundle forming pilus of enteropathogenic *E. coli *[28]. Genes that could encode a type IV pili are found within the genome of Schu S4 [14,29]. A role for type IV pili in invasion or intracellular survival has not been defined but a knockout mutant in a potential pilin-encoding gene in a type B isolate is attenuated by a subcutaneous route in mice, and is impaired in its ability to spread to the spleen [30].

Another potential adaptation to survival in the reducing cytosol could be the activity of GGT. This enzyme has a major role in glutathione metabolism [31]. GGT is the first enzyme in the degradation pathway of glutathione, and transfers a gamma-glutamyl moiety from glutathione to amino acids. GGT can also use gamma-glutamyl peptides as substrates in the reciprocal hydrolysis reaction, thus contributing to the synthesis of glutathione. GGT in *Francisella *may also help to control the influx of glutathione from the cytosol. Further studies on these mutants will help define the roles of these gene products in intracellular survival.

## Conclusion

This is the first reported large-scale mutagenesis and screening of a type A strain of *F. tularensis*. We have identified several genes and pathways that are key for the survival, and growth of a type A strain of *F. tularensis *in a hepatic cell line. Conlan *et al *have shown in a mouse model of tularemia that, regardless of the route of entry, the liver appears to be a primary site of pathology, and intracellular bacteria are visible in hepatocytes [15]. Defects in the growth of mutants in pyrimidine and purine biosynthesis pathways suggest that these pathways are important for optimal intracellular growth, but the slow growth of the *pyrB *mutant inside HepG2 cells and in vivo indicates that the bacteria must be able to procure some nucleobases from the host. Several mutants defective in HepG2 intracellular growth were able to grow similarly to wild-type bacteria in a murine macrophage-derived cell line, suggesting that the intracellular environment, resources, and/or compartment available to the bacteria in these two cells types are different. However, the identification of *dsbB *and *ggt *mutants suggests that *F. tularensis *is in contact with the cytosol in HepG2 cells. We also identified a number of novel intracellular growth-defective mutants that have not been previously characterized in other pathogens. Further characterization of these mutants will provide a better understanding of the pathogenicity of *F. tularensis*, and may have practical applications as attenuated vaccines or through the identification of targets for new therapeutics.

## Methods

### Bacterial strains, and growth conditions

*F. tularensis *subsp. *tularensis *Schu S4 was obtained from the Centers for Disease Control, Fort Collins, CO. Schu S4 was grown either on supplemented Muller-Hinton agar (MHA) (Muller-Hinton Broth 21 g/L, Sodium Chloride 5 g/L, protease peptone 10 g/L, Bacto -Agar 16 g/L) or in enriched Trypticase Soy Broth (TSB). Each medium was supplemented with (wt/vol) 0.1% cysteine, 0.1% glucose, 0.25% ferric pyrophosphate and 2.5% horse serum (vol/vol). Rifampin-resistant mutants were grown in the same media but supplied with 25 μg/ml rifampin. Rifampin is not used in the clinical treatment of tularemia. Permission to construct rifampin-resistant forms of Schu S4 was obtained from the CDC. To test for auxotrophic growth, bacteria were grown in Chamberlain's defined medium (CDM) [32] with or without arginine, and/or uracil at 200 μg/ml. All cultures were inoculated from frozen aliquots of the initial stock, and subcultured no more than three times. The LD_50 _of Schu S4 stock in C57/BL6 mice by intranasal, and intraperitoneal routes was 10–100 bacteria. All mice died within 4 to 5 days. All work with Schu S4 was carried out in an approved Biosafety level 3 laboratory.

*E. coli *TransforMax EC100D *pir-116 *(F^- ^*mcrA *D *mrr-hsdRMS-mcrBC*) W80d *lacZ*DM15 D *lacX74 recA1 endA1 araD*139 D (*ara, leu*) 7697 *galU galK *l^- ^*rpsL nulG pir-166 *(DHFR) was purchased from Epicentre (Madison, WI) [33]. *E. coli *was grown in Luria-Bertani (LB) agar or broth, and when appropriate, ampicillin and rifampin were added to final concentrations of 100 μg/ml and 50 μl/ml, respectively.

### Tissue culture cell lines and growth conditions

Human hepatocellular carcinoma cell line HepG2 (ATCC# HB-8065) and murine macrophage cell J774A.1 (ATCC# TIB-67) were grown in low glucose Gibco™ Dulbecco's Modified Eagle Medium (DMEM) or in high glucose DMEM, respectively, and supplemented with 10% FBS. A 96-well plate was seeded with 20,000 cells per well, and grown at 37°C, 5% CO_2 _overnight before infection with Schu S4-derived mutant strains.

### Transposon library construction

The transposon-containing plasmid, pMW1409, was obtained from David Wood, University of South Alabama. The transposon, EZ::TN<*rpsL*^*p*^*Rparr-2*>, was prepared as described by Qin *et al *[13]. Briefly, pMW1409 was digested with *PvuII*, and the fragment containing the transposon was purified by gel electrophoresis and extraction. One μl of the transposon (120 ng/ml) was mixed with 2 μl transposase, purchased from Epicentre^® ^(1 U/ml) and 1 μl glycerol. This "transposome" mixture was incubated at 37°C for 30 min, room temperature for 30 min, then 4°C overnight. Competent *F. tularensis *Schu S4 bacteria were prepared based on the method described in [34]. Briefly, a fresh culture of Schu S4 was spread on an MHA plate, and incubated at 37°C, 5% CO_2 _overnight. The bacterial lawn was harvested into ice-cold 0.2 M sucrose, washed 4 times, then aliquoted (~5 × 10^8 ^bacteria/50 μl per tube). One μl of the transposome mixture was mixed with 50 μl of competent Schu S4, incubated on ice for 10 minutes then transferred into 0.1-cm-gap electroporation cuvette. The electroporation conditions were 2.5kV, 25uF, and 200 Ohms. After electroporation the cells were plated on a warmed MHA plate, and incubated for 37°C for 6 hrs. The bacterial lawn was collected into 1.6 ml TSB/C. Aliquots of 100 μl were spread on MHA with 25 μg/ml rifampin, and incubated at 37°C, 5%CO_2 _for 48 hrs. Single colonies were inoculated into 96 well microtiter plates containing 150 μl TSB/C with 25 μg/ml rifampin. The plates were incubated at 37°C for 48 hrs. Glycerol was then added to a final concentration of 12.5%. The plates were stored at -80°C.

To verify that EZ::TN<*rpsL*^*p*^*Rparr-2*> was present in a rifampin-resistant colony, a portion of the transposon was amplified by PCR from chromosomal DNA using primers that annealed to transposon mosaic ends (ME) (Fig. 1) and to the 3' of *Rparr-2 *gene (Table 1).

### DNA manipulation, PCR and sequencing

Bacterial chromosomal DNA was isolated using the Puregene^® ^DNA purification kit (Gentra systems, Inc., Minneapolis MN). Plasmid DNA was purified using the QIA prep kit (Qiagen, Valencia, CA). PCR products and DNA restriction fragments were isolated by gel electrophoresis, and purified with the QIA quick gel extraction kit (Qiagen, Valencia, CA).

### Determination of the transposon insertion locations

Identification of the transposon insertion sites was accomplished by rescue cloning, or direct sequencing of bacterial genomic DNA using primers BM008 or BM009 (Table 1) as described in [13] and [35]. Briefly for rescue cloning, 4 μg of chromosomal DNA, isolated from each rifampin-resistant mutant, was digested with *Bcl1 *and self-ligated. The self-ligated plasmids were introduced into *E. coli *TransforMax EC100D *pir-116 *by electroporation. Transformants were selected on LB-rifampin plates. The sequence of the DNA flanking the transposon on the rescued plasmids was determined using primers SqFP or SqRp (Table 1, Epicentre) at the DNA Sequencing facility at the University of Virginia. The identity of the disrupted gene was determined by comparing the sequence to the Schu S4 genomic sequence deposited on the NCBI website.

### Southern blot

Two to four μg of bacterial chromosomal DNA was digested with *BclI*, separated on an 0.8% agarose gel, transferred to nylon membrane, and denatured by standard procedures [36]. The probe used for hybridization was synthesized by PCR using primers 5'arr-2 and 3'arr-2, which anneal to the *Rparr-2 *gene of pMW1409 EZ::TN<*rpsL*^*p*^*Rparr-2*> [13]. The probe was labeled with DIG-dUTP using a kit according to the manufacturer's instructions (Roche, Indianapolis, IN). Hybridization was performed overnight at 50°C in the DIG Easy Hyb buffer (Roche, Indianapolis, IN). After hybridization, the membrane was washed twice for 5 min in 2 × SSC, 1% SDS at room temperature, twice for 15 min in 0.1% SSC, 0.1% SDS at 68°C and once in 0.1% SSC for 5 min at room temperature. Probe hybridization was detected using the protocol of the ECL western blotting detection kit (Amersham, Piscataway, NJ) with anti-DIG-POD as the secondary antibody (Roche, Indianapolis, IN).

### Library screening

The protocol for screening the library was based on the method described by Kawula et al [35]. A duplicate of a stored microtiter plate was created by inoculating 20 μl into a new 96 well plate containing 150 μl TSB/C in each well. This plate was incubated at 37°C, 5% CO_2_, for 48 hrs, which resulted in 0.3 to 0.5 OD_595 _of bacteria in each well. Five μl of the culture was added into another 96 well plate in which 20,000 HepG2 cells per well had been seeded 12 hrs earlier. The multiplicity of infection (MOI) ranged from around 75 to 125 bacteria to 1 host cell. These plates were centrifuged at 1000 × g for 8 min. After incubation at 37°C, 5% CO_2_, for 2 hrs, fresh media with gentamicin was added to a final concentration of 50 μg/ml. The plates were incubated at 37°C, 5% CO_2 _for 24 hrs. After incubation the media was discarded and the cells were lysed by the addition of 150 μl TSB/C containing 0.1% sodium deoxycholate for 5 min at 37°C. Each lysate was transferred to a MHA plate with a 48-pin replicator and incubated for 48 hrs.

### Invasion and intracellular replication assay

HepG2 or J774A.1 cells were seeded into 24 well plates (2 × 10^5 ^cell/well) and incubated at 37°C, 5% CO_2_, for 12 hrs. Before the addition of bacteria, the actually number of HepG2 cells per well was determined by releasing the cells with trypsin from three wells of the 24 well-plate and calculating the number of cells in each well. Bacteria were added at an MOI of 50:1. The plate was centrifuged at 1000 × g for 8 min and then incubated for 1 hr at 37°C, 5% CO_2_. Fresh media containing 50 μg/ml gentamicin was then added. For invasion assays plates were incubated for 1 hr at 37°C, 5% CO_2_; for intracellular replication assays they were incubated for 2–72 hrs. At the end of the incubation time the wells were lysed by the addition of sodium deoxycholate to a final concentration of 0.1% in TSB/C. The number of bacteria was determined by plating dilutions of the lysates on MHA plates. These intracellular growth assays were repeated at least three times.

### Mouse infections

Six to eight week old C57BL/6 mice (Jackson Labs, Bar Harbor, ME) were lightly anesthetized, and then inoculated with bacteria in a volume of 20 μl by an intranasal route. Bacteria were grown overnight in TSB/c broth to a density of 1 × 10^9 ^CFU/ml, and stored in aliquots at -80°C. For inoculation the bacteria were thawed and diluted in sterile saline. The number of bacteria in an inoculum was verified by plating on cysteine heart agar plates supplemented with horse serum. All animals were monitored daily for morbidity and mortality. Animals were euthanized under anesthesia when signs of irreversible mortality were evident.

## Competing interests

The author(s) declare that they have no competing interests.

## Authors' contributions

AQ performed all of the experiments described in this paper, contributed to their design and analysis, and helped to draft the manuscript. BJM conceived of the study and experimental design, contributed to data analysis, and prepared the manuscript. Both authors read and approved the final manuscript.

## Figures and Tables

**Table 1:** Oligonucleotides used in this study

**Name**	**Sequence (5'-3')**	**Source**
SqFP	GCCAACGACTACGCACTAGCCAAC	Epicentre
SqRP	GAGCCAATATGCGAGAACACCCGAGAA	Epicentre
5'RParr-2	TAAGGAGGTATCATATGGTAAAAG	D. Wood^1^
3'RParr-2	GTCTAGAGGATCCTTAATCTTC	D. Wood^1^
BM007	GTTGGCTAGTGCGTAGTCGTTGGC	This study
BM008	GTTGGCTAGTGCGTAGTCGTTGGC	This study
BM009	CGTTAAACATGAGAGCTTAGTACG	This study

**Table 2:** Genes essential for intracellular growth of *F.tularensis *SCHU S4 in Hep G2 cells

**Strain Name**	Locus tag^1^/Genbank protein Accession # (*)	**Gene Name (putative function)**
**Nucleotide metabolism**		
BJM1011	FTT1663/YP_170570	carA (carbamoyl-phosphate synthase small chain)
BJM1012	FTT1664/YP_170571.1	carB (carbamoyl-phosphate synthase large chain)
BJM1001	FTT1665/YP_170572.1 (2X)	pyrB (aspartate carbamoyltransferase)
BJM1014	FTT0204/YP_169264.1	purA (adenylosuccinate synthetase)
BJM1015	FTT1720c/YP_170622.1 (3X)	purL (phosphoribosylformylglycinamidine synthase)
BJM1018	FTT0203c/YP_169263.1 (3X)	purH (bifunctional purine biosynthesis protein)
BJM1020	FTT1721c/YP_170623.1 (2X)	purF (amidophosphoribosyltransferase)^2^
**Amino acid transport and metabolism**		
BJM1002	FTT0588/YP_169611.1	aroA (3-phosphoshikimate 1-carboxyvinyl transferase)
BJM1021	FTT1181c/YP_170144.1	ggt (gamma-glutamyl transpeptidase)
**General function prediction**		
BJM1003	FTT1244c/YP_170202.1	yfiO (conserved hypothetical lipoprotein)
BJM1022	FTT0129/YP_169196.1	major facilitator super family (MFS) transport protein
BJM1004	FTT0056c/YP_169131.1	major facilitator super family (MFS) transport protein
**Inorganic ion transport metabolism**		
BJM1024	FTT1490/YP_170425.1	Na+/H+ antiporter
**Cell wall/membrane biogenesis**		
BJM1023	FTT1234/YP_170192.1	choloylglycine hydrolase family protein
BJM1026	FTT1236/YP_170192.1	hypothetical protein
BJM1028	FTT1103/YP_170079.1	conserved hypothetical lipoprotein
**Lipid metabolism**		
BJM1013	FTT1762c/YP_170653.1	acetyltransferase protein
**Posttranslational modification/protein turnover**		
BJM1025	FTT1310c/YP_170263.1	hflB (ATP-dependent metalloprotease)
BJM1005	FTT0107c/YP_169177.1 (3X)	dsbB (disulfide bond formation protein)

^1^Locus tag:

^2^This mutant was previously identified in *F. tularensis novicida *.

(*) Number of times a mutant in this ORF was isolated [11].

**Table 3:** Invasion and intracellular survival of selected Schu S4 mutants in J774.1^1^

**Strain**	**No. of intracellular CFUs recovered from cells at 24 hrs.**
SCHU S4	3.00 × 10^7 ^± 0.346
BJM1025 (hflB)	1.80 × 10^7 ^± 0.200 p < 0.0065*
BJM1004 (MFS)	1.35 × 10^7 ^± 0.0866 p < 0.0013*
BJM1005 (dsbB)	8.33 × 10^5 ^± 0.0577
BJM1026 (hypothetical)	2.32 × 10^5 ^± 1.29
BJM1001 (*pyrB*)	1.80 × 10^8 ^± 0.0643
BJM1002 (*aroA*)	2.18 × 10^8 ^± 0.387
BJM1003 (*yfiO*)	1.17 × 10^8 ^± 0.135
BJM10012 (*carA*)	1.14 × 10^8 ^± 0.867
BJM10015 (*purL*)	4.03 × 10^7 ^± 0.802
BJM10018 (*purH*)	2.70 × 10^7 ^± 0.100
BJM1024 (Na^+^/H^+ ^transporter)	1.12 × 10^8 ^± 0.872

^1^J774.1 cells were infected with bacteria at an MOI to 50:1 After 2 hrs the media was replaced with media containing gentamicin. Sets of three wells were lysed 24 hrs later, diluted, and spread on MHA plates to determine the number of intracellular colony forming units (CFUs). Each time point represents the average of three wells.

* Compared to Schu S4
